# The Mechanical Environment Modulates Intracellular Calcium Oscillation Activities of Myofibroblasts

**DOI:** 10.1371/journal.pone.0064560

**Published:** 2013-05-14

**Authors:** Charles Godbout, Lysianne Follonier Castella, Eric A. Smith, Nilesh Talele, Melissa L. Chow, Adriano Garonna, Boris Hinz

**Affiliations:** 1 Laboratory of Tissue Repair and Regeneration, Matrix Dynamics Group, Faculty of Dentistry, University of Toronto, Toronto, Ontario, Canada; 2 Laboratory of Cell Biophysics, École Polytechnique Fédérale de Lausanne (EPFL), Lausanne, Switzerland; University of Ottawa, Canada

## Abstract

Myofibroblast contraction is fundamental in the excessive tissue remodeling that is characteristic of fibrotic tissue contractures. Tissue remodeling during development of fibrosis leads to gradually increasing stiffness of the extracellular matrix. We propose that this increased stiffness positively feeds back on the contractile activities of myofibroblasts. We have previously shown that cycles of contraction directly correlate with periodic intracellular calcium oscillations in cultured myofibroblasts. We analyze cytosolic calcium dynamics using fluorescent calcium indicators to evaluate the possible impact of mechanical stress on myofibroblast contractile activity. To modulate extracellular mechanics, we seeded primary rat subcutaneous myofibroblasts on silicone substrates and into collagen gels of different elastic modulus. We modulated cell stress by cell growth on differently adhesive culture substrates, by restricting cell spreading area on micro-printed adhesive islands, and depolymerizing actin with Cytochalasin D. In general, calcium oscillation frequencies in myofibroblasts increased with increasing mechanical challenge. These results provide new insight on how changing mechanical conditions for myofibroblasts are encoded in calcium oscillations and possibly explain how reparative cells adapt their contractile behavior to the stresses occurring in normal and pathological tissue repair.

## Introduction

Myofibroblasts play a beneficial role during normal tissue repair by synthesizing, contracting and remodeling the extracellular matrix (ECM) [1]. Conversely, excessive or deregulated myofibroblast activities cause clinical problems by leading to severe fibrotic conditions that affect multiple tissues and organs, such as skin, heart, lung, and liver [2], [3]. Myofibroblasts are characterized by the neo-expression and incorporation of α-smooth muscle actin (α-SMA) into stress fibers, conferring superior contractile activity compared with their precursor cells [4]. Myofibroblast activation from various progenitors depends on the presence of the pro-fibrotic cytokine transforming growth factor-β1 (TGF-β1) [5] and on a stiff ECM [6]. An increase in ECM stiffness leads to up-regulation of α-SMA expression [7]–[10]. This phenomenon has been explained by the fact that higher contraction, mediated by α-SMA, is required to remodel stiffer tissue [6]. However, it is unclear whether additional control mechanisms exist to modulate or fine-tune myofibroblast contraction beyond the expression and stress-fiber localization of α-SMA. In particular, the impact of the mechanical environment on the spontaneous contractile activity of myofibroblasts remains elusive.

We have recently provided experimental evidence that myofibroblasts employ two modes of contraction, acting simultaneously but independently in a lock-step mechanism [11]. Strong (µN) and long-ranging (µm) isometric contraction is regulated by the small GTPase Rho to generate and maintain slack in ECM fibrils [4], [12]. Such stress-released fibrils can then be subject to local remodelling by periodic low-amplitude (∼100 pN) and short-ranged (∼400 nm) contractions controlled by oscillations in the intracellular calcium concentration ([Ca^2+^]_i_) [12]. Spontaneous and periodic oscillations of [Ca^2+^]_i_ occur in cultured fibroblasts and myofibroblasts and are directly correlated with subcellular contractile events measured with the atomic force microscope [11]. The period of [Ca^2+^]_i_ oscillations in low contractile cardiac and subcutaneous fibroblasts is longer than in their highly contractile myofibroblast counterparts [12], [13]. Other studies have demonstrated that cells develop higher isometric forces and intracellular stress in response to greater mechanical feedback (stiffness) from the ECM [14]–[17]. The effect of higher tension on the [Ca^2+^]_i_ oscillatory activity of myofibroblasts regulating subcellular contractions has not been studied.

We hypothesize that the static mechanical conditions of the extracellular environment control the contractile remodelling activity of myofibroblasts by modulating [Ca^2+^]_i_ oscillation frequency. Using [Ca^2+^]_i_ oscillations as an indicator, we analyze the contractile activity of rat subcutaneous myofibroblasts (SCMF) as a function of the mechanics in different cell culture models. We modulate ECM stiffness by seeding SCMF onto two-dimensional silicone culture substrates and into three-dimensional collagen gels of increasing E-modulus. We vary intracellular stress by modifying cell adhesion strength through different surface coating, by restricting cell size by growing myofibroblasts on specific surface areas created by microcontact printing (μCP), and by inhibiting actin polymerization. Our results demonstrate that increasing mechanical stress increases the frequency of [Ca^2+^]_i_ oscillations in SCMF. Our findings help to understand how changing mechanical conditions for myofibroblasts are encoded in calcium oscillations and possibly explain how reparative cells adapt their contractile behavior to the changing stresses in pathological tissue repair.

## Materials and Methods

### Ethics statement

Animals (rats) were used to harvest primary fibroblasts with ethics approval of the Office of Research Ethics, University of Toronto, protocol no. 20009319. Rats were euthanized using carbon dioxide immediately before dissection.

### Cell culture

We isolated rat subcutaneous fibroblasts from full thickness rat dorsal skin explants and cultured cells up to five passages in Dulbecco's modified Eagle's medium (DMEM; Life Technologies, Burlington, ON, Canada), supplemented with 10% fetal bovine serum (FBS; Sigma-Aldrich, St. Louis, MO), and penicillin/streptomycin (Life Technologies). To promote fibroblast-to-myofibroblast activation, we added TGF-β1 (2 ng/ml; R&D Systems, Minneapolis, MN) for 4–6 days to the culture medium. Cells were then split and grown in presence of TGF-β1 for another two days according to the experimental conditions. For live microscopy, we used homemade observation chambers closed at the bottom by a glass coverslip (#0; Karl Hecht Assistent, Altnau, Switzerland) or, when specifically stated, by a plastic coverslip (Sarstedt, Montréal, QC, Canada).

### Two- and three-dimensional cultures substrates with tuned elastic modulus

To modify extracellular ECM stiffness, we used two-dimensional substrates made of silicone with Young's elastic modulus (E-modulus) tuned to 5 kPa (normal skin/soft), 15 kPa (early tissue repair/medium stiff) or 50 kPa (fibrosis/stiff) (ExCellness Biotech, Lausanne, Switzerland) [7]. We seeded SCMF at a density of 500 cells/cm^2^ on silicone substrates coated with human plasma fibronectin (FN, 2 μg/cm^2^; Millipore, Billerica, MA). As a three-dimensional model, we used collagen type I gels (BD Biosciences, Mississauga, ON, Canada) at concentrations of 1.0, 1.5, 2.0, and 2.5 mg/ml dissolved in 0.02 N acidic acid. Collagen solutions were kept on ice prior to inducing polymerization with neutralizing NaOH. Neutralized collagen was then gently mixed with SCMF suspensions to obtain a final cell concentration of 75,000 cells/ml. The collagen/cell mix was finally pipetted onto the 37°C pre-warmed plastic coverslip of home-made observation chambers and polymerized at 37°C for 1 h. Culture medium was added to the gels that remained attached to plastic coverslips during the culture period of 2 days.

The elastic modulus of cell-free gels was measured using an indentation device mounted to the stage of a Nikon Eclipse TE300 microscope as described in detail elsewhere [18]. Briefly, the indentation device consisted of a μN-resolution tensiometric force probe adapted from the surface tension measurement apparatus of a Langmuir monolayer trough (MicroTrough X, Kibron Inc, Helsinki, Finland). We used a blunt-ended cylindrical tungsten wire as force probe with 300-µm radius, hanging from a digital microbalance and z-controlled with a 157-nm resolution micromanipulator (Micromanipulator 5171, Eppendorf AG, Hamburg, Germany). After calibration, the probe was lowered to register initial point of contact with the collagen gel, which was indented in incremental steps of 10 μm. The decrease in voltage collected by the Filmware software package (Kibron) was converted into a decrease in force measured by the probe, corresponding to an increase in back-force generate by the elastic gel. From here, the elastic modulus was calculated by employing a standard Hertz model [19]. The gel was indented at least three times on one spot over at least three different regions of one gel. We performed three independent readings for each concentration of collagen gels to calculate mean values ± SD.

### Modulation of intracellular mechanical stress

To modify intracellular stress, we first seeded SCMF onto coverslips coated with 2 μg/cm^2^ FN, 0.5 μg/cm^2^ poly-L-lysine (PLL; Sigma-Aldrich), and 5.0 μg/cm^2^ PLL. In contrast to FN that allows integrin binding, PLL provides nonspecific cell binding through electrostatic interactions and limits focal adhesion formation [20]. Second, we controlled cell spreading area by μCP of FN, creating square islands with areas of 100–10,000 µm^2^ on plastic coverslips [7]. Non-printed areas were passivated for cell adhesion with 0.1 mg/ml PLL(20)-graft[3.5]-poly(ethylene glycol)(2) (PLL-g-PEG; Susos, Dübendorf, Switzerland) and SCMF were seeded at a density of 1,000 cells/cm^2^. For this set of experiments, cells were kept in culture for only one day to preserve optimal shape and distribution. In this experimental series only, we stimulated [Ca^2+^]_i_ oscillations with 50 nM endothelin-1 (ET-1, Sigma-Aldrich), as growth on isolated islands reduced spontaneous [Ca^2+^]_i_ oscillations. A series of independent experiments indicated that the lower cell density and larger spacing between cells was causal for the lack of spontaneous oscillations on square islands (data not shown). Third, we inhibited actin polymerization using Cytochalasin D (15 μM; Sigma-Aldrich).

### Imaging and analysis of [Ca^2+^]_i_ oscillations

To visualize [Ca^2+^]_i_ changes, we incubated cells for 45 min with 3 μM Fura-2 AM (Life Technologies) in F-12 medium containing 10% FBS, 20 mM HEPES and 0.25% pluronic acid (Sigma-Aldrich). When plastic coverslips were used as substrate, we incubated cells with 20 μM Fluo-4 AM (Life Technologies) since plastic is autofluorescent at the excitation wavelengths of Fura-2. Next, we washed cells for 15 min with F-12 medium containing 10% FBS and 20 mM HEPES, before recording [Ca^2+^]_i_ dynamics at 37°C on an inverted microscope (Axiovert S100TV, Carl Zeiss, Toronto, ON, Canada) in a humidified atmosphere. The microscope was equipped with a polychromatic Xenon light source (Polychrome IV; TILL Photonics, Gräfelfing, Germany), a high numerical aperture objective (Fluar 10X, NA 0.5) and a charge-coupled device camera (C4742-95; Hamamatsu, Bridgewater, NJ).

Cells incubated with Fura-2 were successively excited at 340 nm and 380 nm every 5 s and pairs of frames were recorded at 510 nm for 15 min, using OpenLab 3.0.6 software (Perkin Elmer, Waltham, MA). Changes in [Ca^2+^]_i_ were calculated over regions of interest covering the entire cells and expressed in arbitrary units as fluorescence ratio  =  Em_340_/Em_380_, as defined in [13]. When using Fluo-4 as Ca^2+^ indicator, excitation and emission wavelengths were 488 nm and 515 nm, respectively. Fluo-4 fluorescence ratio was expressed by F/F_0_ (fluorescence/fluorescence at rest). We calculated the mean period of regular [Ca^2+^]_i_ oscillations and we compiled data of each group into histograms. Distributions were fitted using MatLab software (MathWorks, Natick, MA) and we verified the goodness of the fit with the EasyFit software (MathWave Technologies). For experiments performed with Cytochalasin D, we recorded fluorescence for two 15 min bouts intercalated by a 15 min period during which we added the drug and allowed it to act. We compared [Ca^2+^]_i_ oscillations before and after addition of Cytochalasin D for the same cell.

### Immunofluorescence, microscopy, and quantitative image analysis

Samples were fixed with 3% paraformaldehyde for 10 min, washed with PBS and then permeabilized with 0.2% Triton X-100 for 5 min. We applied primary antibodies against α-SMA (mouse IgG2a, clone SM1, a kind gift of Giulio Gabbiani, University of Geneva, Switzerland), vinculin (mouse IgG1; Abcam, Cambridge, MA), and FN (rabbit IgG, Sigma-Aldrich). We used secondary antibodies goat anti-mouse IgG Alexa Fluor 568 (Life Technologies), goat anti-mouse IgG1 FITC, goat anti-mouse IgG2a TRITC (Southern Biotechnology, Birmingham, AL), and goat anti-rabbit TRITC (Sigma-Aldrich). We used phalloidin Alexa Fluor 488 or 568 (Life Technologies) to stain F-actin and DAPI (Sigma-Aldrich) to label nuclear DNA. Wide-field images were acquired with a 20x objective (Plan Apochromat, NA 0.6) mounted on an inverted microscope (Axiovert 135 M; Carl Zeiss) equipped with a digital camera (C10600 Orca-R^2^; Hamamatsu). Images of collagen gels were acquired in reflection mode using an inverted confocal microscope (DM IRE2; Leica Microsystems, Concord, ON, Canada) equipped with a laser-scanning head (TCS SL) and a 40x oil immersion objective (HCX PL APO, NA 1.25–0.75). Figures were assembled with Adobe Photoshop CS4 (Adobe Systems, San Jose, CA).

Collagen densities, focal adhesion area, and cell spreading area were quantified using Fiji imaging software (Image J, NIH) on confocal images produced from vinculin-, F-actin-, and DAPI-stained SCMF. Focal adhesion lengths were extracted after image thresholding based on vinculin signal, size exclusion of noise pixels, image binarization, and particle analysis for the long axes of best fit ellipses. Total cell area was similarly extracted from F-actin-staining using the area parameter in the particle analysis function and normalized to the number of nuclei in the same image field. Three independent experiments were analyzed with n_cells_≥50 respectively. To quantify the density of collagen gels, single optical sections were taken using confocal reflection microscopy (40×, zoom 2×) with identical settings for all images. The section planes were selected to be 200–600 µm above the support cover slip and to include the nucleus of one cell in the image field with no apparent neighbors. The signal (collagen) density was calculated with a custom routine written in Fiji macro language. Briefly, the integrated density was determined using the measure particle function after applying a mask to the background-subtracted and auto thresholded-image. Values obtained from cell-containing regions were corrected for densities obtained from cell-free regions of the same gel. Per condition, 5 images were analyzed and three independent experiments were performed.

### Statistical analysis

When applicable, data are presented as mean ± standard deviation (SD) or standard error of the mean (SEM). We assessed differences between groups with an analysis of variance (ANOVA) followed by a post-hoc Tukey's multiple comparison test and we set the significance level at p = 0.05. For experiments with Cytochalasin D, we performed a two-tailed paired t-test. Differences were considered to be statistically significant with p≤0.05.

## Results

### Increasing ECM stiffness enhances [Ca^2+^]_i_ oscillation activity

**Figure 1:**
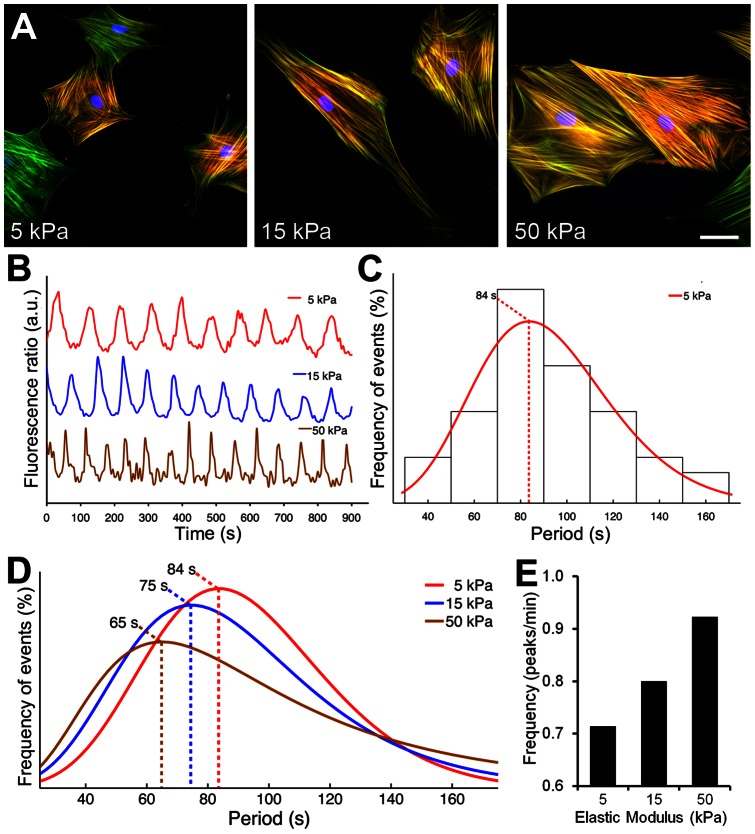
Increasing the E-modulus of silicone substrates increases [Ca^2+^]_i_ oscillation frequency. SCMF were cultured on FN-coated silicone substrates produced with E-moduli of 5, 15, and 50 kPa for 2 days. A) Cells were immunostained for α-SMA (red), F-actin (green) and nuclei (blue). Scale bar = 50 μm. B) Representative fluorescence ratios (Em_340_/Em_380_) were recorded over time on Fura-2-loaded cells of each stiffness group. C) The dominant periods of regular oscillations were determined and pooled into a histogram that was fitted following a generalized extreme value distribution. D) [Ca^2+^]_i_ period distribution fits of 5 kPa, 15 kPa and 50 kPa groups are displayed and maxima highlighted with dotted lines (n_exp_≥14, n_cells_≥44). E) Period distribution fit maxima were translated into oscillation frequency (peaks/min) and expressed as a function of the Young's E-modulus of silicone substrates.

To test the impact of extracellular mechanical stress on [Ca^2+^]_i_ oscillations in myofibroblasts, we first seeded SCMF on silicone substrates of different E-moduli. Substrate E-moduli were tuned to model the mechanical aspects of connective tissues that are either soft/normal (5 kPa), under repair (15 kPa) or fibrotic/stiff (50 kPa) [7]. SCMF cultured on soft substrates exhibited less pronounced stress fibers and were generally smaller than cells grown on stiff substrates (Fig. 1A). In all experiments, we loaded SCMF with fluorescent [Ca^2+^]_i_ indicators. Changes in fluorescence over time, representing spontaneous [Ca^2+^]_i_ oscillations in single cells were recorded (Fig. 1B) and the mean oscillation periods were determined for each cell. All mean periods were compiled in histograms (Fig. 1C), which were fitted to determine the distribution maxima. The distribution fits of SCMF grown on 5 kPa, 15 kPa and 50 kPa substrates exhibited maxima at 84 s, 75 s, and 65 s, respectively (Fig. 1D). Hence, oscillation frequencies ([Ca^2+^]_i_ peaks/min), increased with increasing substrate E-modulus (Fig. 1E).

**Figure 2:**
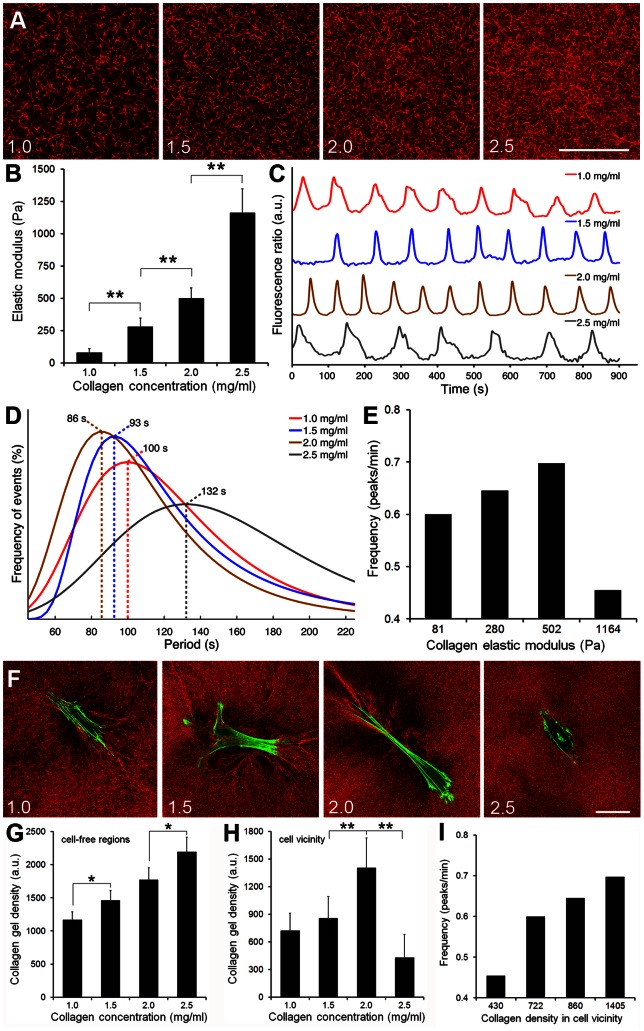
The E-modulus of collagen gels modulates [Ca^2+^]_i_ oscillation frequency. SCMF were grown in gels of 1.0, 1.5, 2.0, or 2.5 mg/ml collagen for 2 days. A) Confocal reflection microscopy imaging of cell-free gels demonstrates collagen fiber density. B) The Young's E-modulus of cell-free gels was measured using a micro-indentation approach. C) Representative fluorescence ratios (F/F_0_) were recorded over time on Fluo-4-loaded cells grown in collagen gels. D) [Ca^2+^]_i_ period distribution fits are displayed and maxima highlighted with dotted lines (n_exp_ = 18–41, n_cells_ = 60–93). E) Period distribution fit maxima were translated into oscillation frequency (peaks/min) and expressed as a function of the Young's E-modulus of collagen gels. F) Myofibroblasts in collagen gels were stained after 2 days for F-actin (green) and collagen ECM was overlaid with confocal reflection imaging (red). Scale bars = 50 μm. The collagen density was measured by applying an integrated density function to confocal images of gels either in G) cell-free regions of the gels or H) in the vicinity of SCMF. I) [Ca^2+^]_i_ oscillatory frequencies are expressed as a function of measured collagen densities (Fig. 2I). (n = 3; mean±SD, **p≤0.01).

To further evaluate the influence of substrate stiffness on [Ca^2+^]_i_ oscillations in a three-dimensional model, we assessed SCMF grown in collagen gels of different E-modulus. Collagen density and stiffness of cell-free gels increased with increasing collagen concentration, as demonstrated by confocal reflection microscopy (Fig. 2A) and measuring the Young's E-modulus (Fig. 2B). Fitted distributions of the mean [Ca^2+^]_i_ oscillation periods recorded on SCMF (Fig. 2C) peaked at shorter periods for higher collagen densities, with 100 s for 1.0 mg/ml, 93 s for 1.5 mg/ml and 86 s for 2.0 mg/ml (Fig. 2D). Hence, in this collagen concentration range, [Ca^2+^]_i_ oscillation frequencies increased with increasing collagen E-modulus (Fig. 2E). However, SCMF grown in highly dense (2.5 mg/ml) collagen gels exhibited a large period distribution with maximum at 132 s, corresponding to a low frequency of 0.45 peaks/min (Fig. 2D, E). To explain this unexpected result we assessed cell morphology and collagen organization degree. Whereas SCMF attained a bipolar shape, formed stress fibers and organized collagen in 1.0–2.0 mg/ml gels, cells were unable to spread and effectively remodel dense 2.5 mg/ml collagen gels (Fig. 2F). Hence, restriction of cell spreading by highly dense gels likely impacted on the ability of SCMF to mechano-sense. The collagen density in cell-free regions of the gels increased with collagen concentration when measured by applying an integrated density function to confocal gels images (Fig. 2G). Consistent with the morphological observation, the degree of collagen organization in the vicinity of SCMF was 1.7–3.2-times higher in 1–2 mg/ml than 2.5 mg/ml collagen gels (Fig. 2H). Increasing collagen density in the vicinity of cells correlated with higher [Ca^2+^]_i_ oscillatory frequencies (Fig. 2I).

### Reducing intracellular stress by modulating SCMF adhesion and spreading area decreases [Ca^2+^]_i_ oscillation activity

**Figure 3:**
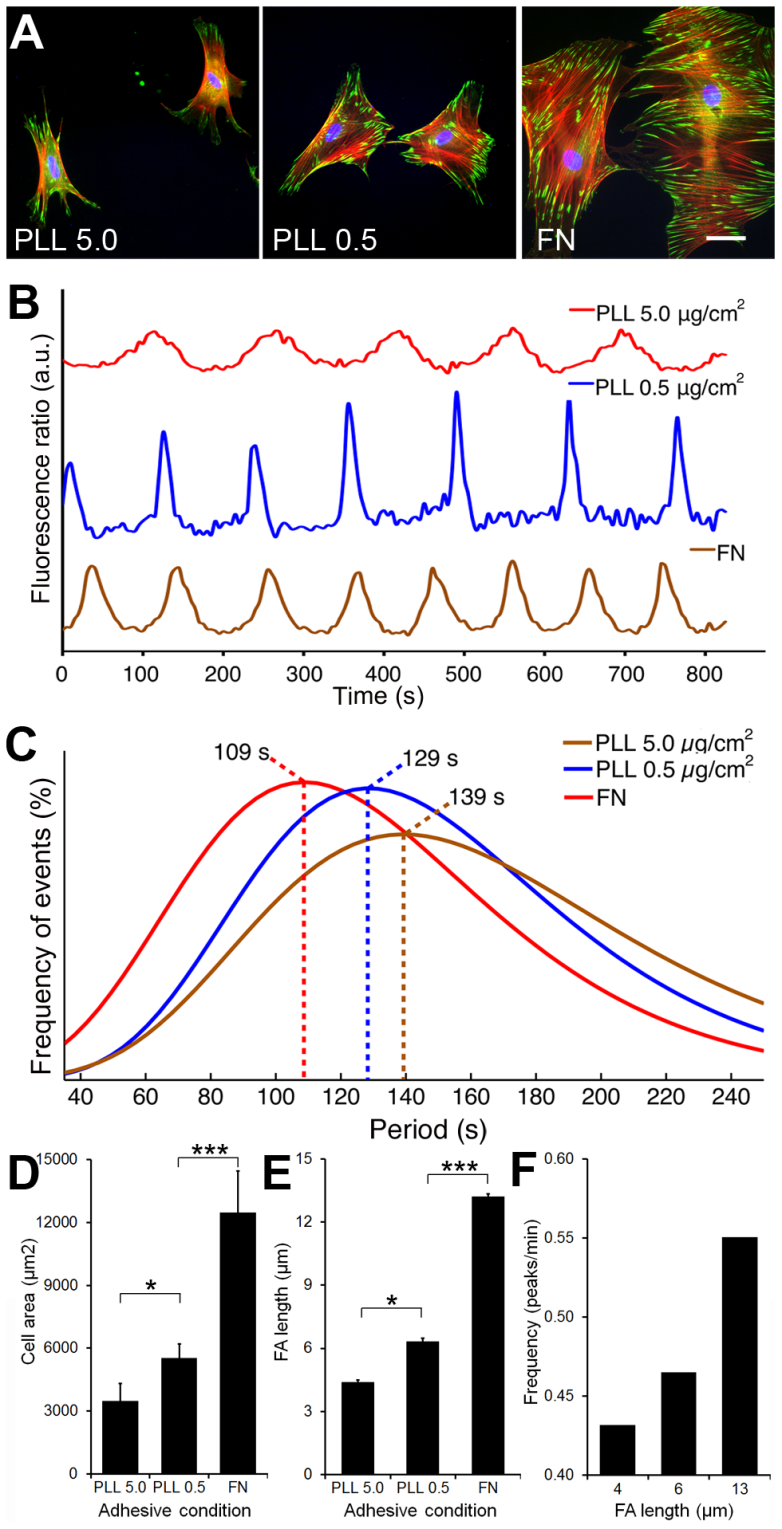
Decreasing cell adhesion decreases [Ca^2+^]_i_ oscillation frequency. SCMF were grown for 2 days on glass coverslips, coated with PLL at 5.0 μg/cm^2^, 0.5 μg/cm^2^, or with FN. A) Cells were immunostained for F-actin (red), vinculin (green) and nuclei (blue). Scale bar = 50 μm. B) Representative fluorescence ratios (Em_340_/Em_380_) were recorded over time on Fura-2-loaded cells. C) [Ca^2+^]_i_ period distribution fits are displayed and maxima highlighted with dotted lines (n_exp_ = 29–34, n_cells_ = 68-93). D) SCMF area (n = 3; mean±SD) and E) the length of vinculin-positive focal adhesions were quantified from fluorescence staining (n = 3; mean±SEM, *p≤0.05, ***p≤0.001), F) Period distribution fit maxima were translated into oscillation frequency (peaks/min) and expressed as a function of the mean SCMF focal adhesion lengths on differently adhesive substrates.

**Figure 4:**
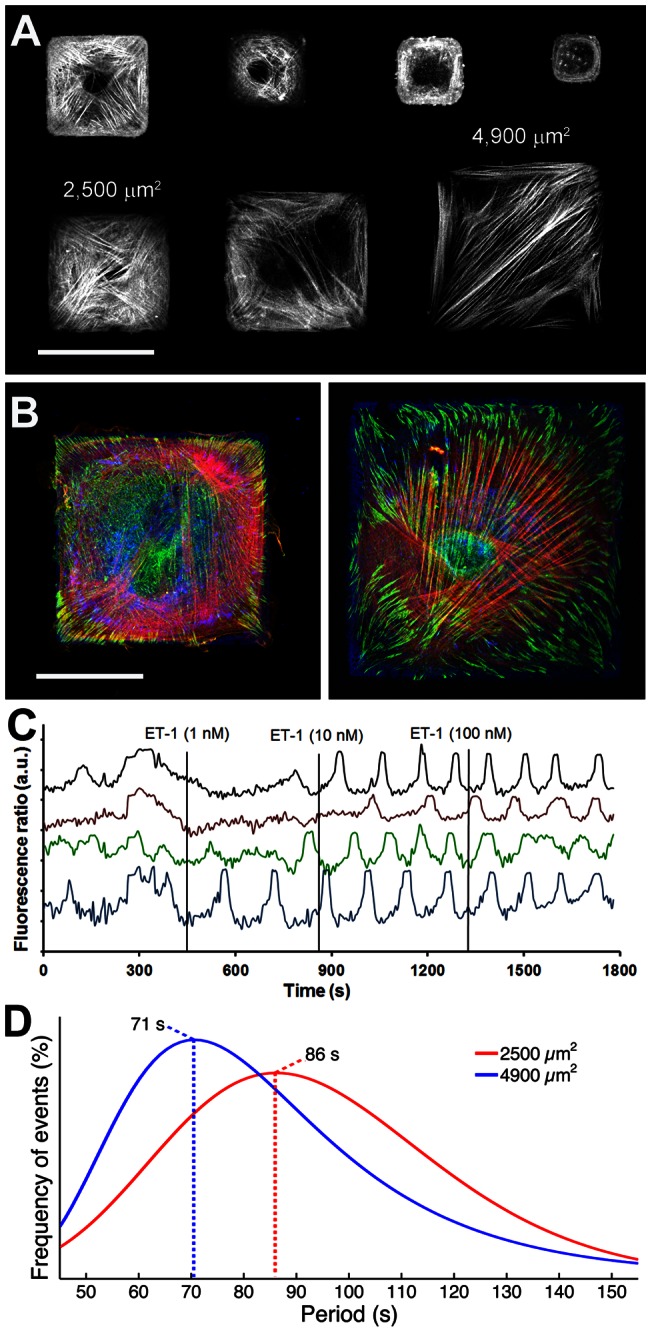
Restricting cell size decreases [Ca^2+^]_i_ frequency. A) SCMF were grown on microcontact-printed FN square islets of 100–10,000 μm^2^, immunostained for F-actin, and imaged by confocal microscopy. A composite was produced by stitching images from different cells on the same substrate, containing all square sizes. Scale bar = 500 μm. B) Cells spreading on FN islets of 2,500 and 4,900 μm^2^ were immunostained for F-actin (red), vinculin (green), and FN (blue). Scale bar = 250 μm. C) Representative fluorescence ratios (Em_340_/Em_380_) were recorded over time on Fura-2-loaded cells, stimulated with increasing concentrations of endothelin-1 (ET-1). D) Distribution fits of [Ca^2+^]_i_ oscillation periods are displayed for cells grown on 2,500 and 4,900 μm^2^ islands (n_exp_ = 18–25, n_cells_ = 24–29) and treated with 50 nM ET-1.

To reduce intracellular stress without chemical interference, we modulated integrin-mediated adhesion of SCMF to planar substrates by coating with FN (highly adhesive), 0.5 μg/cm^2^ PLL (medium adhesive), or 5.0 μg/cm^2^ PLL (low adhesive) [20] (Fig. 3A). When [Ca^2+^]_i_ profiles were analyzed for SCMF (Fig. 4B), we obtained [Ca^2+^]_i_ oscillation period distributions with maxima at 109 s on FN, 129 s on 0.5 μg/cm^2^ PLL, and 139 s on 5.0 μg/cm^2^ PLL (Fig. 3C). A number of previous studies have demonstrated that both, cell spreading area and focal adhesion lengths directly correlate with the level of intracellular cell stress [7], [21]–[24]. Together with increasing stress fiber formation (Fig. 3A), the average surface area of SCMF increased with increasing substrate adhesiveness from 3,470±837 μm^2^ on 5.0 μg/cm^2^ PLL, 5,520±673 μm^2^ on 0.5 μg/cm^2^ PLL, and 12,472±1991 μm^2^ on FN (Fig. 3D). Similarly, the average length of vinculin-positive focal adhesions increased from 4.41±0.08 µm on low adhesive substrates to 13.21±0.13 µm on high-adhesive substrates (Fig. 3A, E). Using focal adhesion size as an indicator of intracellular stress, increasing intracellular stress by higher cell adhesion strength resulted in higher [Ca^2+^]_i_ oscillatory frequencies (Fig. 3F).

Next, we modulated intracellular stress by dictating the spreading area of SCMF using microcontact-printed FN islets of defined dimensions [17], [24]–[26]. Lower levels of stress were indicated by reduced stress fiber formation and focal adhesion size in SCMF on smaller islands compared to larger islands (Fig. 4A, B). We selected small islands of 2,500 µm^2^ and large islands of 4,900 µm^2^ to perform [Ca^2+^]_i_ oscillation experiments (Fig. 4). Cells on islands smaller than 2,500 µm^2^ were highly variable in their ability to form stress fibers and islands exceeding 4,900 µm^2^ frequently hosted more than one cell (unpublished data). Because isolated growth on spaced islands prevented spontaneous [Ca^2+^]_i_ oscillations in SCMF, we provoked [Ca^2+^]_i_ oscillations using 50 nM ET-1 in these experiments only (Fig. 4C). SCMF restricted to a surface area of 2,500 µm^2^ exhibited a maximum at 86 s in their [Ca^2+^]_i_ oscillation period distribution fit (Fig. 4D). SCMF that were allowed to spread to a larger surface area of 4,900 µm^2^ exhibited a maximum at 71 s. Collectively, these data demonstrate that increasing intracellular stress by increasing cell adhesion and cell spreading increased the [Ca^2+^]_i_ frequency of cultured SCMF.

### Depolymerizing F-actin decreases [Ca^2+^]_i_ oscillation activity

**Figure 5:**
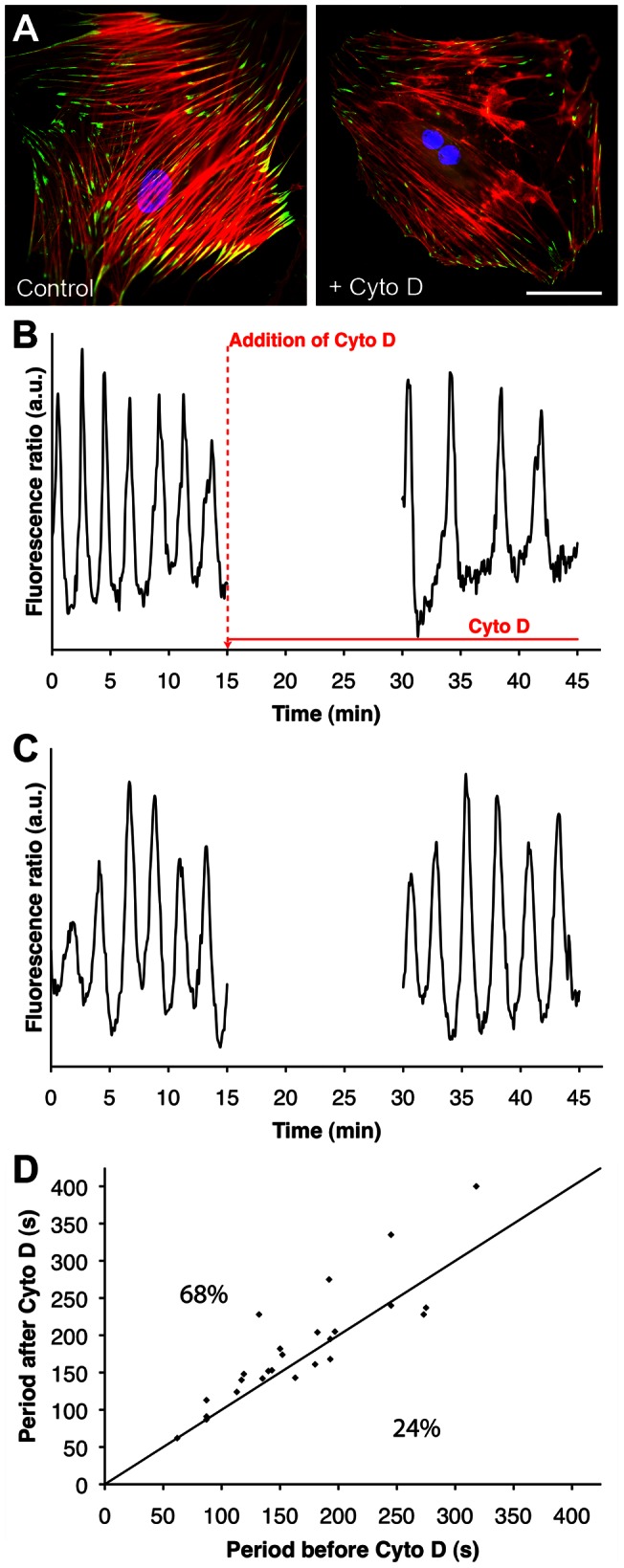
Disrupting actin stress fibers decreases [Ca^2+^]_i_ oscillation frequency. SCMF were grown for 2 days on FN-coated coverslips. A) Cells fixed before (left panel) and 30 min after Cytochalasin D treatment were immunostained for F-actin (red), vinculin (green) and nuclei (blue). Scale bar = 50 μm. B) Representative fluorescence ratio (Em_340_/Em_380_) of a Fura-2-loaded cell over time is shown. Fluorescence was recorded for 15 min before and 15 min after 30 min treatment with Cytochalasin D (15 μM) or vehicle (DMSO) only (C). D) [Ca^2+^]_i_ oscillation period was calculated before Cytochalasin D treatment and plotted against the period after treatment for the same cell. Any point above the diagonal indicates a period decrease after addition of the drug (n_exp = _9, n_cells_ = 25).

Finally, we assessed the role of an intact actin cytoskeleton in regulating [Ca^2+^]_i_ oscillations in SCMF by depolymerizing F-actin using Cytochalasin D (Fig. 5A). In this series of experiments, we added the drug (Fig. 5B) or vehicle only (Fig. 5C) during [Ca^2+^]_i_ recording of single cells and quantified the oscillation periods before and after treatment. To visualize changes, the mean period before drug treatment was plotted against the period after treatment for every cell in bivariate plots (Fig. 5D), as described earlier [13]. For points lying on the diagonal (8% of all cases), the periods before and after Cytochalasin D addition were identical, i.e. the drug had no effect. In 68% of cells, the oscillation periods increased by up to 73% of initial values following Cytochalasin D treatment, indicated by the accumulation of data pairs above the diagonal. In only 24% of cells, [Ca^2+^]_i_ oscillations were unchanged or moderately accelerated by 2 to 16% over control. Only in the cell group with increased periods after treatment the changes were significantly different over controls (p = 0.04). Hence, destruction of the actin cytoskeleton led to an overall decrease in the [Ca^2+^]_i_ oscillation activity of SCMF.

## Discussion

Myofibroblast contraction contributes to normal wound healing and is instrumental in the development of fibrotic contractures [6], [27]. The activation of low contractile precursor cells into highly contractile myofibroblasts is hallmarked by the neo-expression of α-SMA and only occurs with sufficiently stiff ECM [7], [11], [21], [28]–[34]. Incorporation of α-SMA into stress fibers augments fibroblast contraction by 2-4-fold [4], [8], [35], [36] and thus adapts fibroblast contraction to the increasing stiffness of ECM under remodelling. However, to date it was unknown whether and how α-SMA-positive myofibroblasts are able to ‘fine-tune’ their contractile activity to further increasing levels of environmental stress. Previously, we demonstrated that periodic [Ca^2+^]_i_ peaks are directly followed by subcellular contractile events in myofibroblasts [11]. Based on this correlation, we here analyzed [Ca^2+^]_i_ oscillation periods as an indicator for the contractile activity of SCMF in response to varying extracellular and intracellular mechanical stress. We demonstrate that growing SCMF on silicone substrates and in collagen gels of increasing E-modulus, as well as augmenting intracellular stress by manipulating cell adhesion and spreading all increase the [Ca^2+^]_i_ oscillation frequency. Conversely, eliminating stress by disrupting actin stress fibers resulted in the reduction of [Ca^2+^]_i_ oscillation frequencies. Collectively, our data demonstrate that the level of extracellular mechanical challenge modulates oscillatory [Ca^2+^]_i_ dynamics and may thus control the subcellular contractile activity of myofibroblasts.

Ca^2+^ is an important second messenger in transducing mechanical signals. A number of reports have demonstrated Ca^2+^ influx over locally and globally stretched fibroblast membranes (reviewed in [12], [37]–[41]). The level of applied stress is not only coded in the amplitude of single [Ca^2+^]_i_ elevations but in the period/frequency of periodic [Ca^2+^]_i_ oscillations. Straining human gingival fibroblasts on stretchable culture membranes triggered regular and persistent [Ca^2+^]_i_ oscillations [42]. This effect was dependent on intact actin filament bundles and stress-activated ion channels and was abolished by addition of Cytochalasin D and gadolinium [42]. Further, in agreement with our findings, the frequency of spontaneous [Ca^2+^]_i_ oscillations in human mesenchymal stem cells was shown to increase with increasing E-modulus of polyacrylamide gel culture substrates [43]. In this study, the small GTPase RhoA was identified as the main regulatory element whereas the integrity of actin filaments seemed to play no role [43]. This apparent independence of [Ca^2+^]_i_ frequency from the actin organization differs from our observation that disrupting actin stress fibers with Cytochalasin D increased the [Ca^2+^]_i_ oscillation period. However, our data are supported by other studies showing that the tensile state of the actin filament system controls Ca^2+^ activities in various cell types [37], [44], [45]. High levels of stress fiber formation and contraction appear to result in a higher probability for the opening of mechanosensitive Ca^2+^ membrane channels [37], [46]. Consistently, we have previously shown that mechanosensitive channels regulate the entry of extracellular [Ca^2+^]_i_ in SCMF [13].

One possibility to experimentally modulate stress fiber formation and intracellular tension is to culture cells on substrates with tunable E-modulus. The formation of contractile stress fibers is gradually increasing with increasing substrate stiffness in fibroblastic cells [7], [9], [11], [32], [47], [48] and development of cell force is higher on stiff and lower on soft culture substrates [14]–[17], [49]–[52]. Using two-dimensional silicone substrates and three-dimensional attached collagen gel cultures, we demonstrate that conditions that reduced stress fiber formation resulted in reduced [Ca^2+^]_i_ activity in SCMF. Others have shown that the basal and maximal [Ca^2+^]_i_ levels and the amplitude of [Ca^2+^]_i_ transients during cardiomyocyte contraction are amplified by growth on micro-post arrays with an effective E-modulus of 15 kPa compared to 3 kPa [53]. Amplification of [Ca^2+^]_i_ oscillations was further reported in endothelial cells migrating from soft to stiff regions of substrates with a stiffness threshold [37]. The [Ca^2+^]_i_ oscillation frequency possibly plays a role in sensing of ECM mechanical properties and transduction into biochemical signals. Recent works demonstrated that cell-ECM stiffness testing can be performed by very localized and spatially restricted contractions [54], which are in the range of the subcellular contractile events measured in myofibroblasts [11]. Such subcellular contractile units would likely persists after treatment with Cytochalasin D and explain why depolymerization of F-actin dampens but not completely eliminates [Ca^2+^]_i_ oscillations in the presented cases.

Focal adhesions are pivotal elements to develop and perceive stress from the ECM [55]. Growth on micropatterned, low-adhesive, and low E-modulus substrates reduces the size of focal adhesions and concomitantly decreases the development of intracellular tension in myofibroblasts [7], [21], [56]. Limiting the size of focal adhesions and formation of stress fibers using substrate coatings that inhibit integrin binding (PLL) in our experiments reduced the [Ca^2+^]_i_ oscillation frequency in SCMF. Similarly, restricting SCMF spreading to small FN islands produced small focal adhesions, fewer stress fibers and lower [Ca^2+^]_i_ oscillation frequency. These findings are consistent with the observation that the forces developed by cells increase with larger spreading area, associated with higher cortical stiffness, larger focal adhesions and actin stress fiber formation [17], [24]–[26], [57]. In addition to providing the basis of intracellular stress development as a function of ECM stiffness, focal adhesions localize at the sites of trans-membrane [Ca^2+^]_i_ entry via stress-activated channels to the termini of stress fibers [46]. Subcellular localized changes in [Ca^2+^]_i_ have been previously shown to steer actin/myosin-driven directional cell migration [58]–[62] and possibly contribute to the ability of cells to orient and migrate along ECM stiffness gradients in durotaxis [14], [37], [58], [63]–[66]. In myofibroblasts, stress-induced local entry of [Ca^2+^]_i_ at sites of stress fiber connected cell-cell adherens junctions has been shown to coordinate the contractile activity of contacting cells [13]. It remains to be shown whether a similar local mechanism regulates ECM remodelling specifically at sites of cell-ECM focal adhesions.

Overall, SCMF developing larger focal adhesions, more pronounced stress fibers and higher intracellular stress always displayed faster [Ca^2+^]_i_ oscillations. The [Ca^2+^]_i_ oscillation periods measured for SCMF in our different experimental conditions (50–140 s) are in the same range as those reported for cardiac myofibroblasts cultured on 15 kPa soft silicone substrates (∼90 s), and SCMF (∼50 s) and subcutaneous fibroblasts (∼70 s) cultured on glass [11], [13]. Consistent with our hypothesis that higher mechanical stress leads to higher myofibroblast contractile activity, highly contractile SCMF were shown to exhibit shorter oscillation periods than low contractile subcutaneous fibroblasts [13]. Although we did not directly assess contractile events in the present study, our previous works show that periodic [Ca^2+^]_i_ peaks in myofibroblasts regulate periodic contractile events [11], [13]. These periodic contractions act at the subcellular level in a spatial range of ∼400 nm with forces of ∼100 pN. These subcellular contractions are in contrast to long-lasting (hours), long-ranged (tens of µm), and strong (µN) RhoA/Rho kinase-regulated isometric contractions of the whole cell [4], [11]. Occurrence of weak but regular [Ca^2+^]_i_-regulated contractions is expected to result in incremental ECM remodelling in a ‘lock-step’ or ‘ratchet’ mechanism [12]. Consequently, higher [Ca^2+^]_i_ oscillation frequencies in conditions of higher ECM stiffness will result in more efficient ECM remodelling. Together with the finding of other studies that fibroblasts generate higher isometric forces on stiff than on soft substrates [14]–[17], [51], [52], both modes of myofibroblast contraction appear to be adapted to the mechanical conditions of the cell environment.

Adaptation of myofibroblast contraction to the level of extracellularly imposed stress is physiologically relevant during tissue repair, when remodeling of the ECM by myofibroblasts generates a gradual increase of cell tension over time [7], [8]. Understanding the mechanisms of myofibroblast contraction regulation is thus beneficial to clinically interfere with the healing process. When healing is delayed due to reduced wound contraction, such as in chronic diabetic wounds [67], [68], myofibroblast contraction could be improved by increasing mechanical stress. Indeed, mechanical stimulation through cyclic stretching rescues myofibroblast activation and isometric contraction in human dermal fibroblasts cultured under *in vitro* conditions of poor healing [69]. In clinical applications, negative pressure wound therapy has been shown to support the healing process by inducing mechanical stress in the wound [70]–[73]. Conversely, reducing mechanical stress would be beneficial to improve conditions of excessive wound healing such as hypertrophic scarring or fibrosis. Increased mechanical tension in wounds results in long-lasting hypertrophic scars in mice [74] and upregulates myofibroblast activation in rat and human wound models [8], [75]. Use of a mechanical stress-shielding device reduced the formation of hypertrophic scars in the red Duroc swine when compared to control group and conditions of elevated stress [76]. In other organs, such as lung and kidney, anti-cell contraction drugs are being considered to reduce development of fibrosis [77]–[81]. Whether the positive outcomes of “stress-based therapies” *in vivo* can be related to the modulation of myofibroblast contraction at the single-cell and subcellular level and whether this effect is due to modulation of the calcium regulation pathway remain to be shown.
